# Assessment of a New GC-MS/MS System for the Confirmatory Measurement of PCDD/Fs and (N)DL-PCBs in Food under EU Regulation

**DOI:** 10.3390/foods8080302

**Published:** 2019-08-01

**Authors:** Flavio Antonio Franchina, Eliane Lazzari, George Scholl, Jean-François Focant

**Affiliations:** 1 Molecular Systems Research Unit, Faculty of Sciences, University of Liege, B-4000 Liege, Belgium; 2 Institute of Chemistry, Federal University of Rio Grande do Sul, 90040-060 Porto Alegre, Brazil

**Keywords:** targeted analysis, food contaminants, food safety, tandem mass spectrometry (MS/MS), gas chromatography (GC), method validation

## Abstract

Polychlorodibenzo-*p*-dioxins (PCDDs), polychloro-dibenzofurans (PCDFs), dioxin-like (DL), and non dioxin-like (NDL) polychlorinated biphenyls (PCBs) are currently regulated in food and feed within the European territory (EU 2017/644-771). The confirmatory methods of analysis for checking compliance with maximum levels (MLs) for these involve either the historically-established GC-magnetic sector high-resolution mass spectrometry (GC-HRMS) and, more recently, GC-triple quadrupole mass spectrometry operating in tandem mode (GC-QQQMS/MS). In this study, the performance of a novel triple quadrupole GC-QQQMS/MS system equipped with a programable temperature vaporization (PTV) injector was evaluated for the analysis of regulated PCDD/Fs and PCBs in food and feed. The MS analyzer was equipped with a titanium ionization chamber and a new short collision cell capable to accumulate and eject ions by means of very narrow pulses that allow to minimize the noise and to adapt accumulation times for sensitive multiple reaction monitoring (MRM). The analytical capability of the system was confronted by the strict requirements (selectivity, reproducibility, linearity, quant/qual MRM transitions, accuracy, robustness) set by the EU Regulation for a range of standards, quality control (QC) and food/feed samples. In this respect, the approach showed high precision (1.9–15% relative standard deviation (RSD) at low pg/µL) and accuracy (>80%, except for one hexa-CDD). The quantitative results were also compared to the most used GC-HRMS. In this case, comparable results in terms of single congener concentration basis and total toxic equivalent (TEQ) basis for PCDD/Fs and DL-PCBs were obtained for the QC samples analyzed.

## 1. Introduction

Polychlorodibenzo-*p*-dioxins (PCDDs), polychlorodibenzofurans (PCDFs), and polychlorobiphenyls (PCBs) belong to toxic chemicals and are classified as persistent organic pollutants (POPs) [1]. Because of their lipophilicity and high chemical stability, PCDD/Fs and PCBs can remain in the environment, enter the food chain from environmental media, and accumulate in adipose tissues of higher trophic level organisms including humans [2]. Dietary intake of food contaminated with dioxins and DL-PCBs is the major route of human exposure to these toxic organic pollutants [3]. Consequently, this class of compounds has been regulated in Europe [1], following the Stockholm convention for persistent organic pollutants in 2001, in order to protect human health and the environment. Nowadays, the European Commission requires any food or animal feedstuffs released on the market to be controlled and to comply with maximum levels (MLs) set by precise regulatory documents.

Historically, amongst the methods of analysis, gas chromatography coupled to high-resolution mass spectrometry (GC-HRMS) has been recognized as the confirmatory method for POPs determination. The excellent sensitivity and selectivity of a magnetic sector HRMS meet the demand of ultra-trace level contaminants determination in complex matrices, especially if compared to other MS analyzers such as time-of-flight MS, single quadrupole MS, and quadrupole ion storage tandem-in-time MS [4]. However, because of both significant investment and operating costs of GC-HRMS, efforts have focused on the development of alternative approaches in order to reduce dioxin analysis costs. In addition, recent technological advances in triple quadrupole MS analyzers (QQQMS) have made such analyzers a viable alternative to GC-HRMS, especially when operated in tandem mode for targeted analysis at trace levels [5,6]. The use of QQQMS has been also reported for both untargeted and targeted analysis, exploiting a simultaneous scan/ multiple reaction monitoring (MRM) [7,8,9]. However, the targeted approach is by far the most commonly used.

As a direct consequence, and based on other validations studies [10,11], GC-QQQMS/MS begun to successfully show satisfactory performance as a confirmatory tool for checking compliance with maximum levels, following specific analytical criteria [12]. Starting from 2014, European Regulations laying down methods of sampling and analysis for the EU official control of levels of PCDD/Fs and PCBs in food and feed officially started recognizing the use of GC-QQQMS/MS, in addition to the classical GC-HRMS [13,14]. The latest EU Commission Regulations 2017/644 and 2017/771 [15,16] further confirmed the use of GC-QQQMS/MS, even if the majority of the control laboratories still nowadays relies on GC-HRMS technology.

In this study, the performance of a novel triple quadrupole GC-QQQMS/MS system equipped with a programmable temperature vaporization (PTV) injector was evaluated during a validation study for PCDD/Fs, dioxin and non dioxin-like polychlorinated biphenyls (N)DL-PCBs in food and feed. The MS analyzer was equipped with a titanium ionization chamber and a new short collision cell capable to accumulate and eject ions by means of very narrow pulses that allow to minimize the noise and to adapt accumulation times for sensitive MRM. The analytical capability of the system was evaluated under the strict requirements set by the EU Regulation for the 35 target analytes, on quality control (QC) and food/feed samples. Moreover, a comparison of the GC-QQQMS/MS performance with a routine GC-HRMS method is provided.

## 2. Materials and Methods

### 2.1. Standards and Chemicals

Seventeen PCDD/F congeners, four non-ortho (NO-) substituted PCBs, eight mono-ortho (MO-) substituted PCBs, grouped under the term dioxin-like (DL-) PCBs, and six non-dioxin like (NDL-) PCBs were selected for this study. Representative chemical structures of standards employed are presented in Supplementary Figure S1. For PCDD/Fs and (NO-)PCBs, a six-point calibration curve (Cambridge Isotope Laboratories, Inc., Tewksbury, MA, USA) ranging from 0.05 to 50 pg/µL was used. For the (MO-)PCBs and NDL-PCBs, a nine-point calibration curve (CIL) ranging from 0.4 to 500 pg/µL was used. Native and ^13^C-labeled (isotope dilution method) congeners were presents in these calibration solutions. Solutions of standards and purified extracts were made of nonane (Fluka, Munich, Germany) tested to be contamination free. All the regulated target compounds include the most toxic congeners of the families of PCDD/Fs and PCBs which have a toxic equivalent factor (TEF) assigned by the World Health Organization (WHO) [17].

### 2.2. Samples and Certified Reference Material

The samples included in this study were two quality controls, one certified reference material, and three real samples. Quality controls consisting of pork fat and animal feed matrices (natural contamination from the 1999 Belgian dioxins crisis) were fortified with levels concentration in TEQ [ng WHO_2005_TEQ/kg] spanning two orders of magnitude (0.17 and 2) and (0.95 and 0.7), for PCDD/Fs and DL-PCBs, respectively, while for NDL-PCBs was used the concentration levels of 31 pg/µL and 19 pg/µL. The certified reference material (BCR-607) consisted of milk powder and was purchased from IRMM (Geel, Belgium). The proficiency test samples were palm fat, palm oil, and fish liver oil which were provided by the routine laboratory (CART, Liege, Belgium).

### 2.3. Sample Preparation

Samples were prepared following an accredited ISO17025 procedure. Briefly, sample extractions were performed using accelerated solvent extraction (ASE^TM^ 350, Dionex, Thermo Fisher Scientific, Waltham, MA, USA). A multistep automated clean-up and fractionation procedure was used (PowerPrep^TM^ system, FMS Inc., Watertown, MA, USA) to produce two separate fractions (Fraction 1: MO/NDL-PCBs in hexane/dichloromethane 50:50; Fraction 2: PCDD/Fs and NO-PCBs in toluene). The sample preparation method has previously been described in details [18].

### 2.4. Instrumentation and Measurements

A Jeol (Tokyo, Japan) JMS-TQ4000GC triple quadrupole system equipped with a PTV inlet (Optic-4, GL Sciences, Eindhoven, The Netherlands) was used. The MS analyzer was equipped with a titanium ionization chamber. The volume of injection was set at 4 µL for PCDD/Fs and NO-PCBs standard solutions, and 2 µL for MO and NDL-PCBs, injection at 45 °C (5 s), then ramp of 8 °C/s until 325 °C. The vent time was set at 80 s with vent flow of 100 mL/min; the transfer time was 4 min and split flow 25 mL/min until the end of the analysis. All separations were performed with a VF-5ms 50 m × 200 μm × 0.33 μm (Agilent Technologies Inc., Santa Clara, CA, USA) using a temperature program starting at 60 °C (5 min), ramp at 70 °C/s until 200 °C, 3.2 °C/s until 235 °C (1.5 min), 3.2 °C/sec until 270 °C (10 min), 15 °C/sec until 310 °C (10 min), for a total run of 56 min. Transfer line and ion source (EI, 70 eV) temperatures were held at 250 °C. Helium was used as carrier gas at a flow rate of 1 mL/min. Quadrupoles were at 100 °C and the collision cell was held at 150 °C; nitrogen was used as the collision gas. The MS was operated in MRM mode, with collision energy and transitions optimized for native PCDD/F and PCB congeners using an AutoSRM function. Two MRM transitions (quantifier and qualifier), were extrapolated and optimized for each target. Each transition was derived from a specific precursor ions and distinct product ions. Instrument tuning was performed every fifteen days, using the EI high sensitivity autotune mode, and instrument inspection and maintenance were performed regularly. Retention time update supported by Escrime™ software (v. 3.01, Jeol, Tokyo, Japan) assured that the MRM time windows matched with the target analytes elution, in case of column change or cut for maintenance. Comparative analysis was performed on a GC-HRMS (Autospec Ultima, Waters, Milford, MA, USA), using the same chromatographic conditions as in the GC-QQQMS/MS experiments. Averaged relative response factors (RRFs) were used to calculate the concentrations of the target compounds on both GC-QQQMS/MS and GC-HRMS.

### 2.5. Method Validation Criteria

The criteria for the GC-QQQMS/MS method validation followed the requirements of the recent EU regulation 2017/664-771. Method validation was performed through the assessment of the following figures-of-merit: linearity, limit of quantification, precision, and accuracy. Quantification was performed by using the quantifier MRM transitions, and once the target analytes were reliably identified, the experimental qualifier/quantifier ratio and the elution times were used for confirmatory purposes. For the ion ratio, the tolerance interval specified in the regulation (±15%) was set to confirm each target compound avoiding the risk of integration wrong peaks or interferences.

All congeners were quantified against their own ^13^C-labeled internal standards using isotope dilution. Calibration curves were built for each target analyte and the quantitative values were extrapolated using weighted linear regression. Generally, a higher number of injections (*n* = 8) were carried out at the lowest point of the calibration curve. For the remaining calibration points, *n* = 3 and *n* = 4 injections were performed for the PCDD/Fs and NO-PCBs (fraction 2) and MO-PCBs and NDL-PCBs (fraction 1), respectively.

The relative response factors (RRFs) were calculated at each concentration level and the linearity was estimated based on the RFF consistency and in the determination coefficient (R^2^). The accuracy and instrumental limit of quantification (iLOQ) were assessed for each target compound. Accuracy was expressed in terms of bias % and mean squared error (MSE) and it was measured in standard solutions and in certified reference materials. The iLOQ, which is the “performance-LOQ” was calculated at the lowest calibration point and set by 10 times the standard deviation associated with 8 replicate injections. Finally, precision was expressed as relative standard deviation (RSD %) for the calibration curve levels (*n* = 8 for the lowest calibration point).

To monitor the instrumental performance over time, QC samples (10 in total, injected every 5 days, one pork fat and one animal feed matrices QCs, details in Section 2.2.) were analyzed and the analytical performances monitored over a continuous period of 1.5 months. In the same way, instrumental blanks were included every batch of analysis (circa 18 runs) to exclude any possible source of contamination. As an additional control and to monitor the stability of the response, standard solutions were injected at different concentrations twice a week, and over a period of 2.5 months.

## 3. Results and Discussion

### 3.1. Gas Chromatography-Triple Quadrupole Mass Spectrometry (GC-QQQMS/MS) Method Development

The initial part of the study was devoted to the development of the GC and MS conditions. The chromatographic parameters were adapted from the method for PCDD/Fs and PCBs used in our GC-HRMS routine method. In this context, the EU regulation only requires the chromatographic separation of two hexachlorinated (Hx)CDF isomers, the 1,2,3,4,7,8-HxCDF and 1,2,3,6,7,8-HxCDF, specifying the minimum 25% separation, equivalent to a unit resolution (R_s_ = 1). A multi-ramp oven temperature gradient (details in Section 2.4.), with a slow ramp in the elution zones of the penta-, hexa-, and heptachlorinated PCDD/F isomers, facilitated their chromatographic separation on the 5% phenyl-methyl column.

The PTV injection parameters (solvent vent time, transfer time, initial and ramp temperature, split flow) were optimized initially using alkanes dissolved in nonane (i.e., the same solvent of the samples/standards), with the aim to eliminate the solvent and efficiently transfer the target analytes to the column (data not shown). A cold injection condition (45 °C) was also applied, followed by a fast inlet heating (8 °C/s), assuring at the same time the preservation of the thermo-labile compounds and the efficient elimination of the solvent [19]. Indeed, PCDD/Fs are characterized by high boiling points and no significant losses occurred at 45 °C.

With regards to the MS parameters, attention was devoted to the selectivity and sensitivity of the MRM transitions, depending on precursor/product ion combination and MS cycle time, respectively. The MRM transitions (one quantifier and one qualifier) and collision energy values were finely tuned for the 35 target compounds. For the selection of the qualifier and quantifier transition, an automated software optimization tool was used to select the best (most selective and sensitive) precursor/product ions and collision-induced dissociation (CID) energies (see Supplementary Figure S2). As expected, the loss of COCl^•^ from the molecular ion was the most prominent MRM transitions for PCDD/Fs while the ion with the highest response was [M-2Cl]^+^ for PCBs [11]. The CID energies were tested in a 3 V stepwise manner from 5 to 40 V, and the final MRM transitions and energies are listed in Supplementary Table S1.

### 3.2. Method Validation and Regulatory EU 2017/664-771 Compliance

As mentioned before, the isotope dilution method was used. A six-point calibration curve was constructed for the target PCDD/Fs and NO-PCBs (fraction 2), over the 0.05–50 pg/µL range. For MO-PCBs and NDL-PCBs (fraction 1), a nine-point calibration curve was constructed over the 0.4–500 pg/µL range (See Table 1). Determination coefficients were in the range 0.99999 (2,3,7,8-TCDD)–0.9906 (PCB 153).

The repeatability of PCDD/Fs and all the PCBs was evaluated at a concentration ranging from 0.05–0.5 pg/µL, this corresponding to the lowest calibration point (*n* = 8). As reported in Table 1, the results exhibited good repeatability for all the target compounds, with RSD ≤ 15% (1.9–15% range).

The lowest acceptable calibration point was determined according to the two following criteria. First, the calculated RSDs of the lowest level for all congeners must be ≤15%. Second, the relative difference between the RRF average obtained for all points (including replicates) and the RRF average obtained for only the lowest point must be ≤30%, according to the regulation (this is the “acceptable deviation to the RRF”). As can be observed in Table 1, this criterion was met and the linearity was acceptable within the calibration range. At this point, the lowest calibration level was used to determine the iLOQ, as explained before in Section 2.5. The calculated iLOQs were in the range of 0.028 (2,3,7,8-TCDD)–0.273 (OCDF) pg/µL and 0.057 (PCB 81)–0.774 (PCB 123) pg/µL, for PCDD/Fs and PCBs, respectively (Table 1).

**Figure 1:**
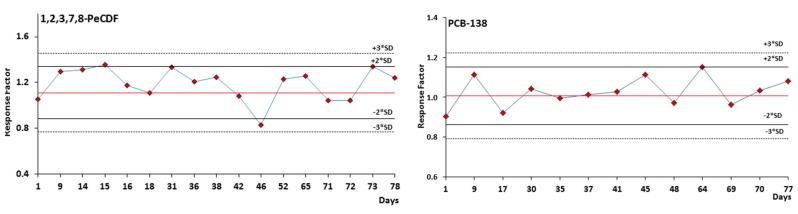
Relative response factors (RRFs) charts for 1,2,3,7,8-PentaChloroDF (PeCDF) and PCB-138 over a 2.5-month period. The lines represent the average RRF, the 99%, and the 95% confidence intervals.

An overtime monitoring of the RRFs stability was carried-out within the entire calibration range and for each target compound. Figure 1 illustrates the RRFs trend for 2 representative compounds (1,2,3,7,8-PeCDF and PCB-138) over a 2.5-month period. As can be observed, the RRF values were inside the 95% confidence range and randomly distributed.

**Figure 2:**
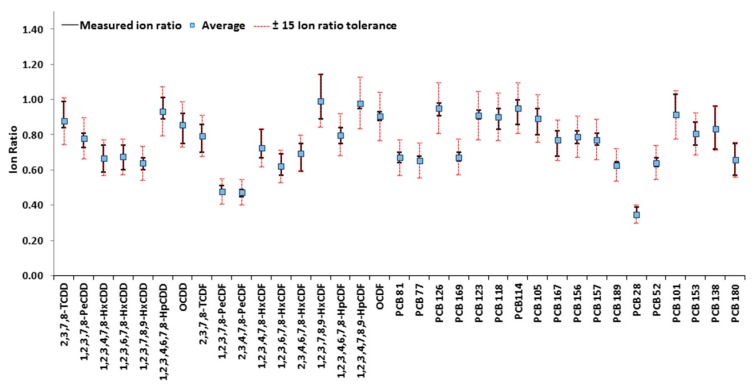
Ion ratio average values for native congeners for all calibration standards (one week of injections, *n* = 25). Black bars: range of minimum/maximum ion ratios; Red dotted bars: 15% tolerance allowed.

In the EU regulations, the relative ion ratio needs to be within the maximum permitted tolerance (±15%) [15,16]. The average (qualifier/quantifier) ion ratio values, derived from the analysis of the calibration solutions and essential for confirmatory methods, are illustrated in Figure 2 (blue dots). On these values, a specific requirement is stated in the legislation, which sets a maximum permitted tolerance of relative ion intensities of ±15% for confirmatory GC-QQQMS/MS methods. The red dotted error bars in Figure 2 represent such a 15% RSD tolerance, and the black error bars illustrate the experimental deviation observed over the entire calibration range. As can be observed, the ion ratio variability met the requirements of the legislation, ranging from 2–15% RSD.

As part of the validation, and in the line of EU requirements, method accuracy for PCDD/Fs and PCBs was evaluated using standard solutions at values close to the ML for animal feed of plant and animal origin (Table 2): the relative bias ranged from −2 to 7.9% among all the target analytes and the MSE values resulted really low (0.009-0.714%).

In addition, a milk certified reference material was analyzed in independent triplicates to demonstrate the efficiency of the method in the presence of a complex fatty matrix. All RSD values were below 15% and the accuracy resulted higher than 80% for all the certified congeners, except for 1,2,3,6,7,8-HxCDD (Table 3). When expressed in TEQ, the accuracy reached 96% (1.99 pg TEQ/g fat measured, 2.07 pg TEQ/g fat certified).

The validation results showed good repeatability and limited bias values, further demonstrating adequate precision and accuracy at the ML values and on the certified food sample.

However, long-term precision and bias studies (12–18 months) would give a better overview of the GC-QQQMS/MS performance.

### 3.3. Quality Controls and Performance Comparison with a Routine Gas Chromatography-High Resolution Mass Spectrometry (GC-HRMS) Method

**Figure 3:**
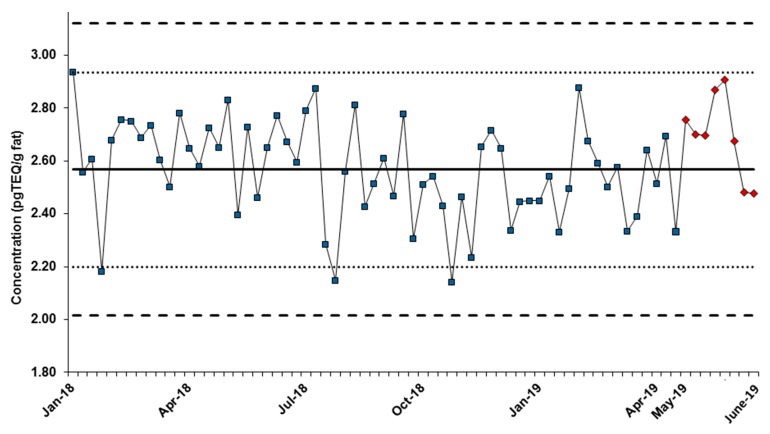
Quality control chart for pork fat routine quality control (QC) samples (sum of polychloridibenzo-p-dioxins (PCDD/Fs) and dioxin-like polychlorinated biphenyls (PCBs) expressed in toxic equivalent (TEQ)). Chart made of routine gas chromatography-high resolution mass spectrometry (GC-HRMS) measurements, with gas chromatography triple quadrupole mass spectrometry (GC-QQQMS/MS) data for the five last points on the right (red dots). Dotted lines represent 95 and 99% confidence intervals (×2 and ×3 SD).

Quality controls, consisting of a pork fat and animal feed samples, were routinely extracted and injected on the GC-QQQMS/MS system. The normalized pg TEQ/g values obtained for the sum of PCDD/Fs and DL-PCBs were included in the control chart fed with GC-HRMS data, as showed in Figure 3 for the pork fat QC. As can be observed, the values obtained with the GC-QQQMS/MS method fitted nicely inside the confidence interval of the routine method.

In the second QC sample (feed), the sum of PCDD/Fs and DL-PCBs resulted with a +7.8% relative error compared to the GC-HRMS, 0.838 and 0.777 pg TEQ/g, respectively.

**Figure 4:**
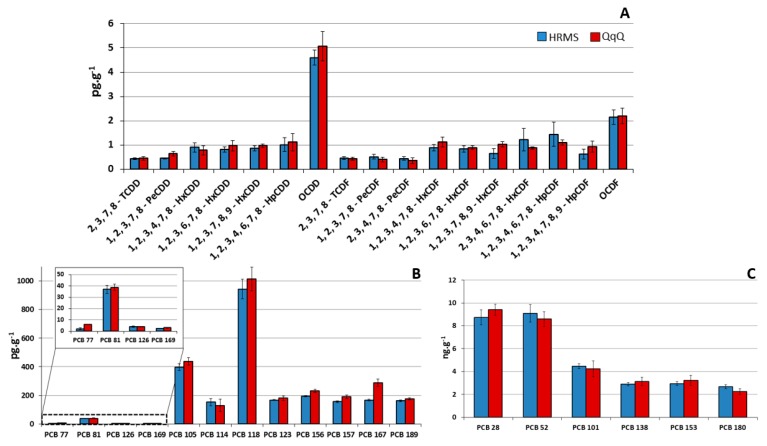
Concentrations and congener profiles of (**A**) polychloridibenzo-p-dioxins/furans (PCDD/Fs), (**B**) non-ortho (NO-) and mono-ortho (MO-) substituted polychlorobiphenyls (PCBs) and (**C**) non dioxin-like (NDL)-PCBs in the pork fat quality control (QC) sample, by using the high resolution mass spectrometry (HRMS) and the QqQ mass spectrometry (MS) methods.

As shown in Figure 4, concentrations and profiles of the congeners are closely related in the GC-QQQMS/MS and the GC-HRMS methods. The bar plot for each target analytes in the animal feed QC sample is reported in Supplementary Figure S3A–C.

Also, a fish liver oil proficiency test sample (EU-RL Halogenated POPs, Freiburg, Germany) was analyzed, which had an assigned value was 0.67 ± 0.128 pg TEQ/g. For this sample, the GC-QQQMS/MS analysis revealed 0.69 pg TEQ/g, confirming again the good accuracy of the method, compliant with the requirements of EU Regulation.

## 4. Conclusions

The use of a novel triple quadrupole GC-QQQMS/MS system equipped with a programmable temperature vaporization injector for the analysis of PCDD/Fs and PCBs was demonstrated to be in line with the last EU requirements for food/feed control. In a 3-month period, the system has been positively tested with standards, quality control, and food/feed samples.

The method showed good sensitivity (iLOQ range: 0.028–0.774 pg/ μL ) and repeatability (1.9–15% range at the lowest point) in analyzing ultra-trace levels of PCDD/Fs and (N)DL-PCBs. In addition, the analysis on the quality control and on the proficiency test samples revealed the results of GC-QQQMS/MS to be comparable to those obtained with the GC-HRMS. These results indicated the validity of this new GC-QQQMS/MS system characterized by a new short collision cell design, as an alternative to the routine GC-HRMS method.

## Figures and Tables

**Table 1:** Calibration curve data and instrumental limit of quantification (iLOQs) for polychloridobenzo-p-dioxins/furans (PCDD/Fs) and polychlorobiphenyls (PCBs).

Native Congeners	Retention Time (min)	Lowest Cali. Point (pg/μL)	Lowest Cali. Point RSD (%)	Highest Cali. Point (pg/μL)	Determination Coef. (R^2^)	Average RRF	Difference (%) RRF (Lowest Point)-RRF (All)	iLOQ (pg/μL)
**PCDDs**								
2,3,7,8-TCDD	29.41	0.05	1.9	10	0.99998	1.35	8.7	0.028
1,2,3,7,8-PeCDD	34.61	0.05	15	10	0.9999	1.07	4.5	0.046
1,2,3,4,7,8-HxCDD	41.11	0.1	11.6	20	0.99996	1.16	22.7	0.168
1,2,3,6,7,8-HxCDD	41.3	0.1	15	20	0.9993	1.18	−3.6	0.094
1,2,3,7,8,9-HxCDD	41.76	0.1	8.3	20	0.99999	1.19	6.3	0.103
1,2,3,4,6,7,8-HpCDD	45.5	0.1	15	20	0.99998	1.38	−25.5	0.06
OCDD	49.97	0.25	12.3	50	0.9999	1.04	8.3	0.164
**PCDFs**								
2,3,7,8-TCDF	28.81	0.05	11.4	10	0.9998	1.24	−12.7	0.032
1,2,3,7,8-PeCDF	32.99	0.05	10.9	10	0.99999	1.09	1.8	0.051
2,3,4,7,8-PeCDF	34.24	0.05	10.2	10	0.99997	1.1	6.4	0.033
1,2,3,4,7,8-HxCDF	39.33	0.1	9.9	20	0.99999	1.03	−5.2	0.09
1,2,3,6,7,8-HxCDF	39.61	0.1	10.2	20	0.99998	1.07	−3	0.053
2,3,4,6,7,8-HxCDF	40.84	0.1	6	20	0.99999	1.23	7.6	0.066
1,2,3,7,8,9-HxCDF	42.33	0.1	8	20	0.999	1.03	−8.5	0.11
1,2,3,4,6,7,8-HpCDF	44.1	0.1	11.9	20	0.9999	1.29	3.9	0.075
1,2,3,4,7,8,9-HpCDF	46.3	0.1	9.5	20	0.99996	1.2	7.1	0.1
OCDF	50.33	0.25	8.2	50	0.9988	1.13	1.6	0.273
**NO-PCBs**								
PCB 77	24.76	0.5	4.8	20	0.9997	1.1	−5.2	0.118
PCB 81	25.33	0.5	6.5	20	0.9997	1.17	−7.5	0.057
PCB 126	29.72	0.5	4.3	20	0.9999	1.37	−0.3	0.253
PCB 169	34.41	0.5	8	20	0.9999	1.12	−3.2	0.397
**MO-PCBs**								
PCB 123	26.37	0.4	3.7	140	0.9913	1.2	14.1	0.774
PCB 118	26.57	0.4	2.5	140	0.9908	1.26	15.8	0.233
PCB114	27.16	0.4	6.3	140	0.995	1.19	6.6	0.756
PCB 105	27.98	0.4	6.8	140	0.9976	1.25	14.6	0.226
PCB 167	30.68	0.4	5.1	140	0.9926	1.15	15.1	0.121
PCB 156	32.01	0.4	9.2	140	0.9928	1.13	15.5	0.255
PCB 157	32.31	0.4	13.7	140	0.9998	1.21	11.7	0.494
PCB 189	37.16	0.4	10.1	140	0.9998	1.14	14.1	0.119
**NDL-PCBs**								
PCB 28	17.95	0.4	7.5	500	0.9963	0.78	17	0.21
PCB 52	19.23	0.4	8	500	0.9914	1.21	14.3	0.176
PCB 101	23.29	0.4	7.8	500	0.9961	1.31	19.8	0.13
PCB 153	27.64	0.4	14.9	500	0.9906	1.05	−4.9	0.434
PCB 138	29.13	0.4	4.5	500	0.9908	0.98	2.5	0.235
PCB 180	32.92	0.4	12	500	0.9927	0.99	−12.5	0.249

**Table 2:** Bias % and mean squared error (MSE) of the method for polychloridibenzo-p-dioxins (PCDD/Fs) and dioxin-like polychlorinated biphenyls (DL-PCBs) using standards solutions at concentrations near the maximum level (ML) for animal feed.

**plant origins** ng WHO_2005_TEQ/kg							
**PCDD/Fs**	**Regulation**	**Average**	**SD**	**RSD %**	**Target**	**Bias %**	**MSE**
ML	0.75	0.573	0.018	3.21	0.584	−1.88	0.00035
ML/2	0.38	0.126	0.003	2.41	0.117	7.69	0.00009
2ML	1.5	1.166	0.012	1.06	1.168	−0.15	0.00011
**animal origins (fat)** ng WHO_2005_TEQ/kg							
**PCDD/Fs**	**Regulation**	**Average**	**SD**	**RSD %**	**Target**	**Bias %**	**MSE**
ML	1.5	1.72	0.055	3.21	1.75	−1.95	0.00308
ML/2	0.75	0.378	0.009	2.41	0.35	7.87	0.00084
2ML	3	3.52	0.037	1.06	3.5	0.34	0.00115
**plant origins** ng WHO_2005_TEQ/kg							
**DL-PCBs** and **PCDD/Fs**	**Regulation**	**Average**	**SD**	**RSD %**	**Target**	**Bias %**	**MSE**
ML	1.25	1.375	0.014	1.02	1.372	0.28	0.00014
ML/2	0.625	0.654	0.019	2.85	0.665	−1.77	0.00024
2ML	2.5	6.152	0.103	1.67	6.247	−1.52	0.00714
**animal origins (fat)** ng WHO_2005_TEQ/kg							
**DL-PCBs** and **PCDD/Fs**	**Regulation**	**Average**	**SD**	**RSD %**	**Target**	**Bias %**	**MSE**
ML	2	1.961	0.056	2.846	1.996	−1.77	0.00407
ML/2	1	0.493	0.01	2.016	0.47	4.82	0.00048
2ML	4	4.126	0.042	1.017	4.115	0.28	0.00129

**Table 3:** Performance of the method for the certified polychloridibenzo-p-dioxins/furans (PCDD/Fs) congeners present in milk BCR-607 (pg/g fat).

Analytes	Measured Values	SD	RSD (%)	Certified Values *	Accuracy (%)
2,3,7,8-TCDD	0.26	0.025	10	0.25(0.03)	105
1,2,3,7,8-PeCDD	0.85	0.057	7	0.79(0.04)	107
1,2,3,4,7,8-HxCDD	0.34	0.018	5	0.42(0.07)	81
1,2,3,6,7,8-HxCDD	0.65	0.071	11	0.98(0.11)	66
1,2,3,7,8,9-HxCDD	0.28	0.024	8	0.34(0.05)	83
2,3,7,8-TCDF	0.04	0.004	10	0.05(0.03)	82
1,2,3,7,8-PeCDF	0.06	0.005	8	0.054(0.013)	114
2,3,4,7,8-PeCDF	1.49	0.152	10	1.81(0.13)	82
1,2,3,4,7,8-HxCDF	0.91	0.073	8	0.94(0.04)	97
1,2,3,6,7,8-HxCDF	1.06	0.074	7	1.01(0.09)	105
2,3,4,6,7,8-HxCDF	0.97	0.051	5	1.07(0.05)	90

* Uncertainties in brackets.

## Supplementary Materials

The following are available online at https://www.mdpi.com/2304-8158/8/8/302/s1, Figure S1: Chemical structures of the PCDDs, PCDFs, PCBs congeners, Figure S2: Ion intensities graph used for the MRM method development and optimization, Figure S3: Concentrations and congener profiles of (A) PCDD/Fs (B) NO-PCBs and MO-PCBs and (C) NDL-PCBs in the animal feed QC sample, Table S1: MRM transitions, collision energies and retention times of the target compounds and ^13^C IS.
