# Supplementary material for: Assessment of a New GC-MS/MS System for the Confirmatory Measurement of PCDD/Fs and (N)DL-PCBs in Food under EU Regulation
Source: Foods. 2019 Aug 1;8(8):302. doi: 10.3390/foods8080302 (PMC6722952; doi:10.3390/foods8080302)
Supplement: Supplementary file 1 [file foods-08-00302-s001.zip › foods-560258-SI.pdf]

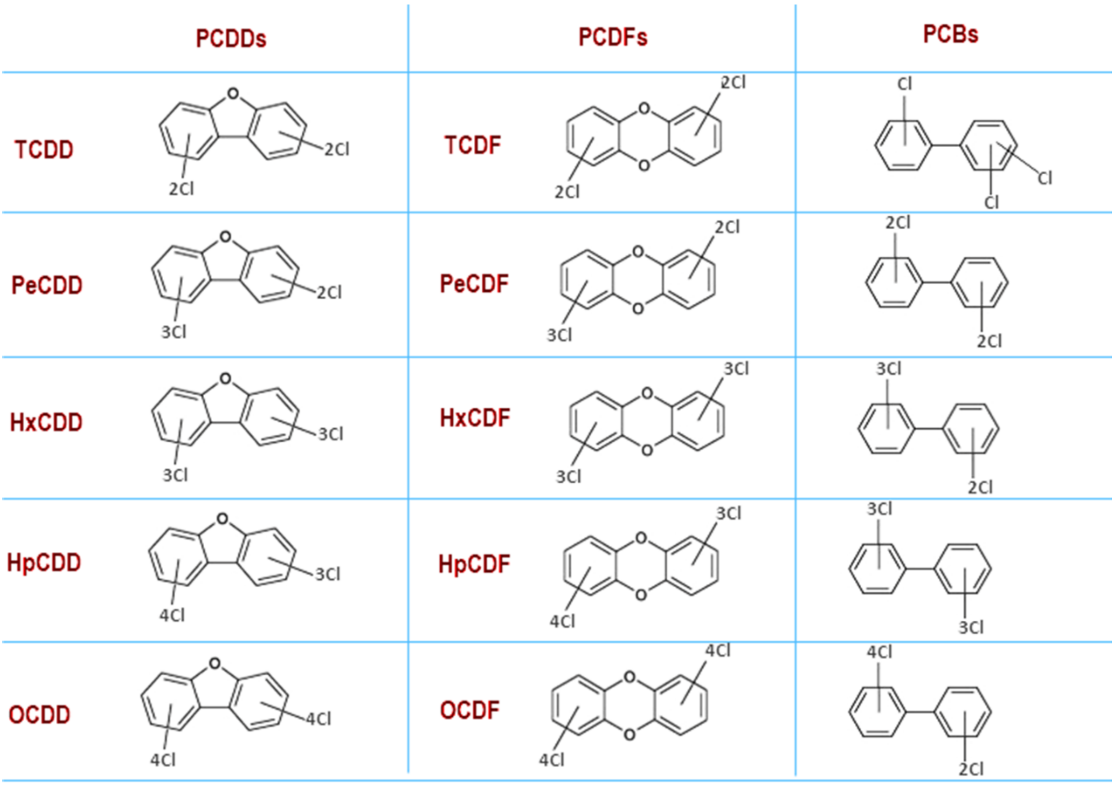

Figure 1. Chemical structures of the PCDDs, PCDFs, PCBs congeners.

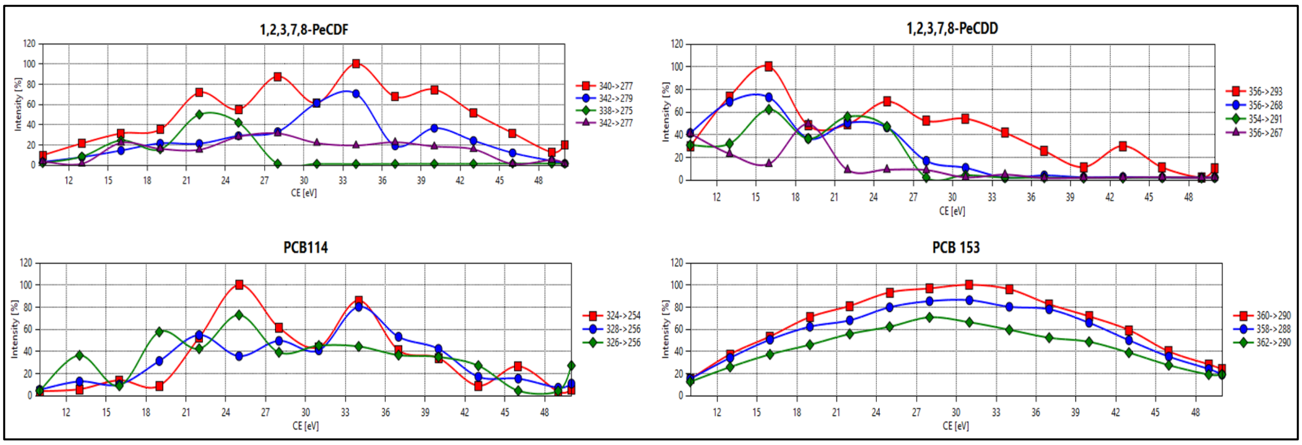

Figure 2. Ion intensities graph used for the MRM method development and optimization.

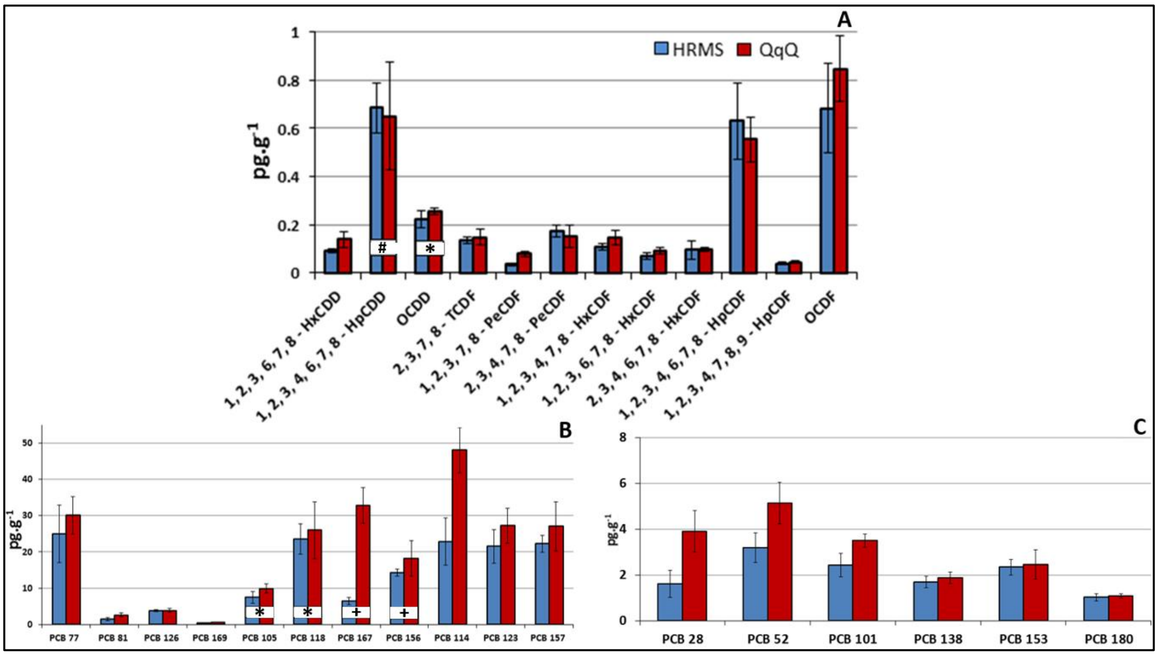

Figure 3. Concentrations and congener profiles of (A) PCDD/Fs (B) NO-PCBs and MO-PCBs and (C) NDL-PCBs in the animal feed QC sample. For scale reasons, the symbols #, +, and * indicate that the quantity was divided by a factor 2, 10 and 50, respectively.

Table S1: MRM transitions, collision energies and retention times of the target compounds and ^{13}C IS.

Congeners	Retention time	Quantifier transition		Qualifier transition	
		Precursor → Product	CE (V)	Precursor → Product	CE (V)
**PCDDs**					
2,3,7,8-TCDD	29.41	322→259	25	320→257	31
^{13}C-2,3,7,8-TCDD	29.42	334→270	25	332→268	31
1,2,3,7,8-PeCDD	34.62	356→293	16	354→291	16
^{13}C-1,2,3,7,8-PeCDD	34.63	368→304	16	366→302	16
1,2,3,4,7,8-HxCDD	41.11	390→328	34	388→325	25
^{13}C-1,2,3,4,7,8-HxCDD	41.12	402→338	34	404→340	25
1,2,3,6,7,8-HxCDD	41.30	390→327	22	392→329	28
^{13}C-1,2,3,6,7,8-HxCDD	41.31	402→338	22	404→340	28
1,2,3,7,8,9-HxCDD	41.76	390→327	22	392→329	22
^{13}C-1,2,3,7,8,9-HxCDD	41.76	402→338	22	404→340	22
1,2,3,4,6,7,8-HpCDD	45.50	424→361	16	426→363	19
^{13}C-1,2,3,4,6,7,8-HpCDD	45.51	436→372	16	438→374	19
OCDD	49.97	460→397	19	458→395	16
^{13}C-OCDD	49.98	472→408	19	470→406	16
**PCDFs**					
2,3,7,8-TCDF	28.77	304→241	34	306→243	25
^{13}C-2,3,7,8-TCDF	28.78	316→252	34	318→254	25
1,2,3,7,8-PeCDF	33.01	340→277	34	342→279	34
^{13}C-1,2,3,7,8-PeCDF	33.02	352→288	34	350→286	34
2,3,4,7,8-PeCDF	34.24	340→277	34	342→279	34
^{13}C-2,3,4,7,8-PeCDF	34.25	352→288	34	350→286	31
1,2,3,4,7,8-HxCDF	39.33	374→311	37	376→313	34
^{13}C-1,2,3,4,7,8-HxCDF	39.34	386→322	37	388→324	34
1,2,3,6,7,8-HxCDF	39.61	374→311	34	376→313	31
^{13}C-1,2,3,6,7,8-HxCDF	39.62	386→322	34	388→324	31
2,3,4,6,7,8-HxCDF	40.84	374→311	28	376→313	31
^{13}C-2,3,4,6,7,8-HxCDF	40.85	386→322	28	388→324	31
1,2,3,7,8,9-HxCDF	42.33	374→311	43	376→313	31
^{13}C-1,2,3,7,8,9-HxCDF	42.34	386→322	43	388→324	31
1,2,3,4,6,7,8-HpCDF	44.10	408→345	34	410→347	34
^{13}C-1,2,3,4,6,7,8-HpCDF	44.11	420→356	34	422→358	43
1,2,3,4,7,8,9-HpCDF	46.30	410→347	34	408→345	43
^{13}C-1,2,3,4,7,8,9-HpCDF	46.31	420→356	34	422→358	43
OCDF	50.33	442→379	34	444→381	43

^{13}C -OCDF	50.34	456→392	43	454→390	43
**NO-PCBs**					
PCB 77	24.76	290→220	25	292→222	28
^{13}C -PCB 77	24.77	302→232	25	304→234	28
PCB 81	25.33	290→220	28	292→222	28
^{13}C -PCB 81	25.34	302→232	28	304→234	28
PCB 126	29.72	324→254	22	326→256	22
^{13}C -PCB 126	29.73	338→268	22	336→266	22
PCB 169	34.41	362→290	28	360→288	25
^{13}C -PCB 169	34.42	374→302	28	372→300	25
**MO-PCBs**					
PCB 123	26.37	324→254	31	326→256	28
^{13}C -PCB 123	26.38	336→266	31	338→268	28
PCB 118	26.57	326→256	31	324→254	25
^{13}C -PCB 118	26.58	336→266	31	338→268	25
PCB114	27.16	326→256	25	324→254	25
^{13}C -PCB114	27.17	338→268	25	336→266	25
PCB 105	27.98	324→254	31	326→256	31
^{13}C -PCB 105	27.99	336→266	31	338→268	31
PCB 167	30.68	360→290	31	358→288	28
^{13}C -PCB 167	30.69	372→302	31	370→300	28
PCB 156	32.01	360→290	28	358→288	28
^{13}C -PCB 156	32.02	372→302	28	370→300	28
PCB 157	32.31	360→290	31	358→288	28
^{13}C -PCB 157	32.32	372→302	31	370→300	28
PCB 189	37.15	394→324	34	396→326	31
^{13}C -PCB 189	37.16	406→336	34	304→334	31
**NDL-PCBs**					
PCB 28	17.95	256→186	22	258→188	22
^{13}C -PCB 28	17.96	268→198	22	270→200	22
PCB 52	19.23	290→220	25	292→222	25
^{13}C -PCB 52	19.24	302→232	25	304→234	25
PCB 101	23.29	326→256	31	324→254	25
^{13}C - PCB 101	23.30	336→266	31	338→268	25
PCB 153	27.64	360→290	31	358→288	31
^{13}C - PCB 153	27.65	372→302	31	370→300	31
PCB 138	29.13	360→290	25	358→288	25
^{13}C - PCB 138	29.14	372→302	25	370→300	25
PCB 180	32.92	394→324	34	396→326	31
^{13}C - PCB 180	32.93	406→336	34	408→338	31
